# Effect of hormone replacement therapy on the bone mass and urinary excretion of pyridinium cross-links

**DOI:** 10.1590/S1516-31802000000100002

**Published:** 2000-01-02

**Authors:** Dolores Perovano Pardini, Anibal Tagliaferri Sabino, Ana Maria Meneses, Teresa Kasamatsu, José Gilberto Henriques Vieira

**Keywords:** Pyridinium cross-links, Hormone replacement therapy, Bone remodeling, Excreção urinária de piridinolinas, Terapia de reposição hormomal, Remodelação óssea

## Abstract

**CONTEXT:**

The menopause accelerates bone loss and is associated with an increased bone turnover. Bone formation may be evaluated by several biochemical markers. However, the establishment of an accurate marker for bone resorption has been more difficult to achieve.

**OBJECTIVE:**

To study the effect of hormone replacement therapy (HRT) on bone mass and on the markers of bone resorption: urinary excretion of pyridinoline and deoxypyridinoline.

**DESIGN:**

Cohort correlational study.

**SETTING:**

Academic referral center.

**SAMPLE:**

53 post-menopausal women, aged 48-58 years.

**MAIN MEASUREMENTS:**

Urinary pyr and d-pyr were measured in fasting urine samples by spectrofluorometry after high performance liquid chromatography and corrected for creatinine excretion measured before treatment and after 1, 2, 4 and 12 months. Bone mineral density (BMD) was measured by dual energy X-ray absorptiometry (DEXA) before treatment and after 12 months of HRT.

**RESULTS:**

The BMD after HRT was about 4.7% (P < 0.0004); 2% (P < 0.002); and 3% (P < 0.01) higher than the basal values in lumbar spine, neck and trochanter respectively. There were no significant correlations between pyridinium cross-links and age, weight, meno-pause duration and BMD. The decrease in pyr and d-pyr was progressive after HRT, reaching 28.9% (P < 0.0002), and 42% (P < 0.0002) respectively after 1 year.

**CONCLUSIONS:**

Urinary pyridinoline and deoxypyridinoline excretion decreases early in hormone replacement therapy, reflecting a decrease in the bone resorption rate, and no correlation was observed with the bone mass evaluated by densitometry.

## resumo

**CONTEXTO:**

A perda óssea acelerada observada na pós menopausa é atribuída a um incremento do turnover ósseo, com predomínio da reabsorção em decorrência da falência ovariana. Ao contrário da formação tem sido difícil de estabelecer um marcador sensível de reabsorção.

**OBJETIVOS:**

Avaliar o efeito da terapia de reposicão hormonal (TRH) na densidade e nos marcadores urinários de reabsorção óssea, piridinolina (PIR) e deoxipiridinolina (D-PIR).

**TIPO DE ESTUDO:**

Estudo coorte de correlação.

**LOCAL:**

Centro universitário de referência.

**PACIENTES:**

53 mulheres menopausadas há 3,6 ± 5 anos, idade mediana de 53 anos.

**VARIÁVEIS ESTUDADAS:**

A excreção urinária de PIR e D-PIR na urina de jejum ,analisada por HPLC e expressa em pmol /mmol de creatinina ,foi avaliada em condições basais e após 1,2,4 e 12 meses de TRH. Realizaram densitometria óssea (DO) em coluna e fêmur antes e após 12 meses de TRH.

**RESULTADOS:**

A DO após TRH foi 4.7%(P<0.0004); 2%(P<0.002); and 3%(P<0.01) maior que os valores basais na coluna , colo do fêmur e trocanter respectivamente. Observou-se um decréscimo dos valores de PIR e D-PIR precoce e progressivo, atingindo 28,9% (P<0.0002) e 42% (P<0.0002) após 1ano de TRH respectivamente. Não houve correlação entre os valores de DO e os de PIR E D-PIR.

**CONCLUSÕES:**

A excreção urinária de PIR e D-PIR constituem um marcador sensível da reabsorção óssea. O decréscimo precoce a torna útil na avaliação da eficácia terapeutica.

## INTRODUCTION

The menopause is associated with an increase in bone resorption rate which may contribute to osteoporosis, for which the main consequence is an increased risk of fractures.^1^ Hormone replacement therapy (HRT) has been shown to be the most efficient prevention and treatment of osteoporosis.^2^ Accelerated postmenopausal bone loss is attributed to an increase in bone turnover, considering both bone formation and resorption due to ovarian failure, with the predominance of resorption.^3,4^

Bone formation may be evaluated through different biochemical markers, such as serum concentration of osteocalcine, alkaline phosphatase and the carboxyl terminal pro-peptide of type I procollagen.^5^ How-ever, it has been difficult to establish a sensitive resorption marker. Most studies on the postmenopause period are based on the determination of urinary hydroxyproline, which is not specific for bone collagen but is a fraction originating from skin collagen, which also decreases in the postmenopausal period.^6^

Urinary pyridinoline (pyr) and deoxypyridinoline (d-pyr) excretion has been used as a new bone resorption marker. Pyr and d-pyr are non-reducible interlinking molecules of bone collagen, not subject to significant metabolism in the body and are released during their degradation. Although collagen is the major component of several connective tissues, pyr is abundant in bone and cartilage, and d-pyr seems to be present in significant amounts only in bone.^7,8^ Therefore, urinary excretion of pyr and d-pyr may reflect the bone matrix degradation process in a sensitive way.

The purpose of this study was to determine the validity of these markers in the early evaluation of the efficacy of hormone replacement therapy in relation to bone resorption in menopausal women.

## METHODS

The procedures that follow were in accordance with the ethical standards of the comittee responsible for human experimentation and with the Helsinki declaration of 1975, as revised in 1983.

### Design

Cohort correlational study.

### Setting

Academic referral center.

### Sample

The study consisted of 53 women with menopause of 3.6 years (SD 5) before the study, and ages of 53 years (SD 5 median 50 years) and body mass index (BMI) of 26 kg/m^2^ (SD 4). They were healthy women without previous use of any hormonal or other medication that could affect bone metabolism. Calcium intake, as assessed by questioning about food, was between 500 and 1000 mg/day in 26% and below 500 mg/day in 74% of the patients. Menopause was defined as amenorrhea for more than 6 months and/or FSH serum levels over or equal to 40 mIU/ L. In 96% of the women the menopause was spontaneous and mean FSH levels were 65.9 mIU/L (SD 27.2).

### Study Procedures

The patients were submitted to standard questioning, physical and gynecological examination and, when included in the study, bone mineral density (BMD) of the column and femur, serum gonadotrophin (LH, FSH), estradiol and urinary pyridinoline and deoxypyridinoline determinations were performed. Then the patients started hormone replacement therapy with 100 mcg/week transdermal estradiol (Estraderm^®^) and 5 mg/day/12days/month oral medroxyprogesterone (Farlutal^®^), except for two patients who had hysterectomies and used only estradiol.

### Main Outcomes

Pyr and d-pyr were assayed in urine after 2 h fasting. Urine samples were collected after 1, 2, 4 and 12 months of HRT for pyr and d-pyr evaluation and BD was repeated after 12 months of HRT. The urine was hydrolyzed and submitted to CF1 cellulose chromatography. The extracted product was separated by HPLC and identified using fluorescein. Pyr and d-pyr results were expressed as pmol/μmol creatinine.^9^ The inter-assay variation coefficient was 12.4% for pyr and 10.8% for d-pyr and the intra-assay was 0.6% for pyr and 6.7% for d-pyr.

Bone densitometry was done using a double energy X-ray absorption apparatus (Lunar Radiation DPX, Madison, WI) with a variation coefficient of approximately 1.5% for both the column and the femoral neck.

Estradiol and gonadotrophin levels were determined by radioimmunoassay.

### Statistical Methods

Paired and non-paired t tests were used to compare variables within the same group at the beginning and after the treatment and to compare variables between the groups, respectively. To evaluate values repeated throughout the experiment, variance analysis was used and complemented with Tukey's contrast test. In order to correlate the two variables we used Pearson's correlation test. The level of significance was set at P < 0.05 (5%). Results shown in the text and tables are expressed as mean and standard deviation.

## RESULTS

After 1 month of HRT d-pyr values already presented a decrease of 34% when compared with the baseline values (P < 0.001), and at 2 months, the pyr decrease was 14% (P < 0.05). The decrease progressed up to 1 year of HRT reaching 28.9% (P < 0.0002) for pyr and 42% (P < 0.0002) for d-pyr (Table 1).

**Table I:** Pyridinium cross-links before and after hormone replacement therapy

Variable	basal	After Treatment			
		1 month	2 months	4 months	1 year
**pyr pmol/umol**	41.4 (17)	35.6 (20)	35.2 (21)*	32.3 (15)**	30.7 (17)***
**d-pyr pmol/umol**	5.0 (3.8)	3.3 (3.9)**	3.4 (4.4) *	4.7 (11.8)	2.9 (5.2)***

*** P < 0.0002;

** P < 0.001;

* P < 0.05 basal vs. after treatment; values in mean and standard deviation.

Regarding body mass, an increase in that of the column, femoral neck and trochanter was observed after 1 year of HRT. BMD values were 4.7% (P < 0.0004); 2% (P < 0.002); 3% (P < 0.01) higher than the baseline values, respectively (Table 2).

**Table 2:** Bone mineral density before and after hormone replacement therapy

Location	BMD•, g/cm^2^	
	basal	1 year
**Lumbar spine**	0.97 (0.18)	1.02 (0.18)***
**Neck**	0.82 (0.13)	0.84 (0.12)**
**Trochanter**	0.70 (0.14)	0.72 (0.13)*

*** P < 0.0004;

** P < 0.002;

* P < 0.01 basal vs. after treatment; values in mean and standard deviation.

• Bone mineral density

On comparing pyr and d-pyr baseline values and those after HRT with BMD values for all analyzed sites, no correlation could be found with pyr. D-pyr correlated with column BMD in the first month of treatment (P < 0.02; r = −0.42).

Before starting HRT we found 21 osteoporotic women (39.6%) (Z score = −2.50 standard deviations as compared with young adults); 8 osteopenic women (15%) (Z score between −1.00 and 2.50 standard deviations as compared with young adults). No correlation between BMD, for either osteoporotic or osteopenic women with baseline pyr and d-pyr levels, was found in these patients.

Baseline pyr and d-pyr values did not correlate with body mass index (BMI), age and time of meno-pause of the patients.

Baseline BMD was correlated with BMI (column P < 0.02, r = 0.49; femoral neck P < 0.03, r = 0.46; tro-chanter P < 0.0001, r = 0.58); age (column P < 0.01, r = −0.31; femoral neck P < 0.04, r = −0.30; trochanter P < 0.01, r = −0.38); time of menopause (column P < 0.01, r = −0.36; femoral neck P < 0.03; r = −0.31; trochanter P < 0.04, r = −0.31).

## DISCUSSION

Estrogenic deficiency of menopausal women leads to an increase in bone resorption, while increase in formation is secondarily increased due to phenomena occurring in parallel.^10^ The rate of bone loss is due to an imbalance between the two processes and it may be expected that a sensitive resorption marker would be able to reflect these changes in bone turnover.

According to previous studies, the menopause leads to a significant increase in urinary pyridinoline and deoxypyridinoline excretion. Uebelhart et al observed an increase of 128% for pyr and 20% for d-pyr (in 24-hour urine samples) when compared with premenopausal women in the age range of 31 ± 6 years,^11^ showing that the resorption process is quite active.

The effect of HRT on the bone mass of menopausal women is mainly due to reduction in resorption, reflecting a decrease in the frequency of activation in new bone remodeling units.^12,13^ In fact, urinary pyr and d-pyr determinations have shown that these substances are quite specific markers of bone resorption. Our findings were that within the first month of HRT in the case of pyr, and the second month for dpyr, a significant decrease could be observed in comparison with the pretreatment values. This reinforces the value of these markers, in addition to demonstrating their early estrogenic effect on bone.

Uebelhart observed that after 6 months of HRT, pyr and d-pyr levels returned to premenopausal values.^14^ However, our findings showed a continuous decrease of 28.9% and 42%, respectively, similar to Hassager's findings.^1^ It is probable that after this length of HRT, stabilization of the resorption process and consequently the marker levels occurs, in accordance with findings by Schlemmer, whose patients were followed-up for 10 years.^15^

Since the postmenopausal bone loss rate is determined by the ratio of formation to resorption phenomena, we would expect that sensitive resorption markers such as pyr and d-pyr would correlate with mineral bone density as assessed by densitometry. However, except for d-pyr in the first month of HRT, which correlated with column BMD, no correlation between markers and body mass could be observed, whether at baseline conditions or after treatment. Other authors have obtained the same results, reinforcing the idea that these markers are not useful for the diagnosis of patients with osteoporosis, but may help in therapeutic monitoring.^14^ In contrast to Schlemmer, who found different pyr and d-pyr values in osteoporotic and osteopenic patients diagnosed by BMD,^15^ our patients showed no difference between the groups with or without osteoporosis and osteopenia. One explanation for this finding is that it may be due to the fact that bone mass measurement, as assessed by densitometry, is made only at some sites such as the column, femur or radius and evaluation of urinary markers reflects collagen degradation of the whole skeleton, including different segments which may present different turnover and bone loss rates.^16^

Most previous studies on urinary pyr and d-pyr excretion refer to alterations observed in the post-menopausal period in comparison with the premenopausal condition.^5,14,17^ There are not many longitudinal studies that extend beyond 6 months of HRT.

## CONCLUSION

We conclude that urinary pyridinoline and deoxypyridinoline excretion decreases during hormone replacement therapy, reflecting a decrease in the bone resorption rate. This decrease is early and is therefore helpful in the evaluation of therapeutic efficacy.
